# Facilitators and Barriers to Uptake of an Extended Seasonal Malaria Chemoprevention Programme in Ghana: A Qualitative Study of Caregivers and Community Health Workers

**DOI:** 10.1371/journal.pone.0166951

**Published:** 2016-11-29

**Authors:** Gifty D. Antwi, Laura A. Bates, Rebecca King, Princess R. Mahama, Harry Tagbor, Matt Cairns, James N. Newell

**Affiliations:** 1 School of Public Health, Kwame Nkrumah University of Science and Technology, KNUST, Kumasi, Ghana; 2 Nuffield Centre for International Health and Development, Leeds Institute of Health Sciences, University of Leeds, Leeds, United Kingdom; 3 Centre for Global Health Research, KNUST, Kumasi, Ghana; 4 Tropical Epidemiology Group, London School of Hygiene and Tropical Medicine, London, United Kingdom; Centers for Disease Control and Prevention, UNITED STATES

## Abstract

**Background:**

Seasonal Malaria Chemoprevention (SMC) is currently recommended for children under five in areas where malaria transmission is highly seasonal. We explored children’s caregivers’ and community health workers’ (CHWs) responses to an extended 5-month SMC programme.

**Methods:**

Thirteen in-depth interviews and eight focus group discussions explored optimal and suboptimal ‘uptake’ of SMC to examine facilitators and barriers to caregivers’ uptake.

**Results:**

There did not appear to be major differences between caregivers of children with optimal and sub-optimal SMC uptake in terms of their knowledge of malaria, their perceptions of the effect of SMC on a child’s health, nor their understanding of chemoprevention. Caregivers experienced difficulty in prioritising SMC for well children, perceiving medication being for treatment rather than prevention. Prior to the study, caregivers had become accustomed to rapid diagnostic testing (RDT) for malaria, and therefore blood testing for malaria during the baseline survey at the start of the SMC programme may have positively influenced uptake. Facilitators of uptake included caregivers’ trust in and respect for administrators of SMC (including CHWs), access to medication and supportive (family) networks. Barriers to uptake related to poor communication of timings of community gatherings, travel distances, absence during SMC home deliveries, and limited demand for SMC due to lack of previous experience. Future delivery of SMC by trained CHWs would be acceptable to caregivers.

**Conclusion:**

A combination of caregivers’ physical access to SMC medication, the drug regimen, trust in the medical profession and perceived norms around malaria prevention all likely influenced caregivers’ level of uptake. SMC programmes need to consider: 1) developing supportive, accessible and flexible modes of drug administration including home delivery and village community kiosks; 2) improving demand for preventive medication including the harnessing of learnt trust; and 3) developing community-based networks for users to support optimal uptake of SMC.

## Introduction

### Background

Although there is evidence of decreasing malaria morbidity and mortality globally, malaria remains an important contributor to child mortality in sub-Saharan Africa [1]. In Ghana, it accounts for 38% of all outpatient illnesses, 35% of hospital admissions and 34% of all deaths in children under five years [2]. Control strategies have included the use of insecticide-treated nets, indoor residual spraying, prompt diagnosis and treatment with artemisinin-based combination therapy (ACT) and intermittent preventive treatment in pregnant women.

In 2012, the World Health Organization (WHO) recommended seasonal malaria chemoprevention (SMC) [3] for the control of malaria in children under five where malaria transmission is highly seasonal, primarily the Sahel sub-region of sub-Saharan Africa [4]. SMC is “the intermittent administration of full treatment courses of an antimalarial medicine during the malaria season to prevent malarial illness” [3] and is delivered up to four times at monthly intervals during the peak malaria transmission period in the area. SMC has been proven to be effective in reducing episodes of uncomplicated malaria and severe malaria by about 75% and is cost-effective and safe [3]. Its delivery by community health workers (CHWs) has been shown to be feasible and to increase uptake compared to clinic-based delivery [5].

### Extended SMC programme

To date, trials of SMC have mostly been conducted in places with a short rainy season, and there has consequently been uncertainty whether SMC might also be beneficial in areas of an extended malaria transmission [6–10]. In response, we undertook a trial of monthly SMC over five months in an area of extended malaria transmission, in the Ashanti Region of Ghana [clinicaltrials.gov identifier: NCT01651416], a predominantly rural area with moderately high malaria transmission throughout the year. Although the extended SMC programme reduced malaria incidence by around 40% during the rainy season, reflecting an important public health impact, this efficacy was lower than seen elsewhere, and coverage of SMC was generally lower than in countries where a shorter SMC programme was used [11].

### Aim

To assess the acceptability of the extended SMC programme and identify facilitators and barriers to caregivers’ uptake of SMC for their children, we conducted a qualitative study alongside the trial. The five objectives were to understand caregivers’ (i) knowledge of malaria to determine whether it affects uptake of SMC, and to examine (ii) perceived effect of SMC on children’s health, (iii) experiences and attitudes concerning the concept of SMC, (iv) experiences and attitudes concerning the regimen of the chemoprevention and in particular the extended period of SMC delivery, and (v) preferred place of administration of SMC. We were particularly interested in factors that may have facilitated caregivers accepting at least four of the five SMC doses (termed optimal uptake).

## Method

### Study setting

The randomised controlled trial (RCT) was conducted in all 13 communities of the Kwaso sub-municipality of the Ejisu-Juaben Municipality of the Ashanti Region of Ghana ([1]. Children aged between three months and five years in July 2012, living in study communities already providing home-based management of malaria (HMM) were enrolled in the study. Between July and November 2012, children were given either five monthly SMC cycles consisting of a single dose of sulfadoxine-pyrimethamine plus amodiaquine given over three days, or matching placebos. During the first day of the first study month, the field team and CHWs held a community gathering to recruit children to the study and administer the first of three daily doses of SMC. Caregivers were required to self-administer the remaining doses of amodiaquine to their children over the following two consecutive days. Over the remaining four subsequent months the study team and CHWs repeated this process of SMC administration. However, for children whose carers did not attend a community gathering (fourth month onwards), CHWs visited children at home to deliver the first daily dose. CHWs also visited the children each month after day three of the dosing regimen to record any side-effects.

CHWs supported many health activities in the community, such as monthly child welfare clinics, national immunization days, and administration of oral rehydration sachets for children with diarrhoea, supervised by the health directorate of their district. CHWs are known to the study communities through a Home Management of Malaria (the HMM programme established in 2007. CHWs within the HMM programme are based at home, or at a known location in their village. Using a standard algorithm the CHWs manage children seeking treatment for fevers, or other symptoms, by immediate referral to a health facility or treated presumptively with artesunate-amodiaquine. Recently, rapid diagnostic tests (RDTs) were introduced to the HMM programme, and case-management of confirmed malaria cases is with artemether-lumefantrine (AL). Children with negative tests are referred to the health centre for further management. No conditions other than malaria were treated by the CHW during the trial period; children with signs of severe disease or where malaria was excluded as a cause of illness were referred to the health facility.

The 13 communities were divided into three groups for the qualitative study: peri-urban communities with a health care facility (HCF), peri-urban communities with no HCF and rural communities with no HCF. Two communities per group were randomly selected for inclusion into the qualitative study. In-depth interviews (IDIs) with caregivers of children who had received the active SMC, and focus group discussions (FGDs) with caregivers of children who had received both active SMC and placebo, took place after the administration of the fifth SMC dose (see Table 1). All participants were female. Caregivers of children enrolled into the SMC trial were predominantly of Ashanti ethnicity (the remainder being of Ewe, Grumani and Grushi ethnicity) and of Muslim or Christian faith.

**Table 1:** Female caregivers and community health workers who took part in In-Depth Interviews or Focus Group Discussions.

Method	Community characteristics	SMC Uptake	No. ID
13 In Depth Interviews with Caregivers	Periurban villages with HCF	≥ 4 doses (optimal)	1A, 2A
	Periurban village with HCF	≤ 3 doses (sub-optimal)	1B, 2B, 3B
	Periurban village without HCF	≥ 4 doses (optimal)	1C, 2C
	Periurban village without HCF	≤ 3 doses (sub- optimal)	1D, 2D, 3D
	Rural village without HCF	≥ 4 doses (optimal)	1E, 2E, 3E
	Rural village with HCF	≤ 3 doses (sub- optimal)	-
6 Focus Group Discussions with Caregivers	Rural village without HCF	Varied uptake	F1, F2, F3
	Periurban village without HCF	Varied uptake	G1, G2
	Periurban village with HCF	Varied uptake	H1
2 Focus Group Discussions with CHWs	Community Health Workers (mixed villages)	-	CHW1, CHW2

### Recruitment and sampling

Uptake was categorised into those participants who received at least four cycles of SMC (hereafter ‘optimal’ uptake, even though it is not ideal for children to miss any of the monthly SMC cycles) and those who received fewer than four cycles (hereafter ‘sub-optimal’ uptake). In each of the six communities, three children with optimal uptake and three children with sub-optimal uptake were randomly selected from each community, using the register list and the random number function in Stata. CHWs in the six communities helped to locate the caregivers of these children and invited them for interview. Thirteen caregivers (seven optimal and six sub-optimal) across all communities presented and consented for interview, however no caregivers with sub-optimal uptake from the 'rural community without HCF' presented on the day of interview, with CHWs reporting they were not available that day (see Table 1).

In addition to the SMC registers, the number of SMC cycles provided to each child was verified using the enrolment card when the caregiver attended the IDI. It was not possible to verify if caregivers had actually administered the two home doses each month, other than by self-report, which may be prone to social desirability bias. The definition of uptake is, therefore, strictly based on administration of the first dose of SMC and delivery of remaining tablets to the caregiver.

One FGD, involving 8–11 caregivers, was held per community. These caregivers were randomly selected from the list of those recruited for the RCT (after eliminating those that had been selected for IDI), irrespective of the number of doses of SMC received by their child. Two further FGDs were conducted with CHWs from all 13 communities involved in the SMC trial. CHWs were distributed between the two groups such that no two persons in the group belonged to the same community.

### Data collection

Data was collected using semi-structured interview guides (developed by GA and RK) that covered the scope of the research objectives. The guides were pre-tested prior to implementation and revised accordingly.

During January to February 2013, data was collected by the field team, comprising three experienced local, female, public health facilitators/interviewers. Two interviewers facilitated the FGDs and the third conducted the IDIs. All IDIs and FGDs were conducted in the local dialect, Twi, at local community centres. IDIs took up to 20 minutes while FGD lasted an average of one hour. Interviews and group discussions were audio–recorded, translated and transcribed into English and proof read by the field team. Transcripts were not returned to the participants for comment or correction before analysis was conducted, due to logistical constraints.

### Data entry and analysis

Two researchers (LB and GA) familiarised themselves with all transcripts before they were uploaded into NVivo v10. Three researchers (LB, GA, and RK) independently open-coded 2 IDIs and 2 FGDs. The codes were then grouped by LB and arranged within an analytical framework structured around the 5 categories that mirrored the pre-defined objectives of the study. This analytical framework was populated through indexing transcripts into the agreed codes. The framework was continually revised during this process by adding or refining the name of existing codes. Changes to the codes were agreed with the study team. The data were then charted into framework matrices to compare the characteristics of and differences between participant groups for each category and to consider connections between categories [12]. Iterative review, reflection and analysis of the matrices revealed a number of themes and subthemes, which are detailed in Table 2.

**Table 2:** Results of the coding and framework analysis.

Categories of codes (mirroring pre-defined objectives)	Codes (used in Nvivo)	Themes (identified through analysis of framework matrix)	Sub-theme (detailed aspects of themes)	Key findings (Interpretation)
Knowledge of malaria	Malaria names	Malaria Literacy		The term ‘malaria’ is often used interchangeably with ‘fever’
	Malaria health effects		Multiple causes of malaria	Confusion over promoted health messages
	Malaria causes		Risk aware	Children are recognised as a vulnerable group
	Malaria seasons		Prevention practices aware	Behaviours reflect CHWs’ health messages
	Malaria vulnerability		Prevention medication unaware	Malaria-related health education may not improve uptake of SMC
	Malaria general prevention in adults or in general			
	Malaria general prevention in children			
	Malaria prevention—IPT experience			
	Malaria prevention—IPT knowledge			
	Malaria treatment seeking behaviour			
Perceived effect of SMC on children's health	SMC good effects on children	Perceived Influence of SMC	Varied uptake and interpretation of side-effects	Uptake varied despite positive health effects
	SMC bad effects on children		Supportive networks (f)	Supportive assurances may counteract negative influences
	SMC effects on caregivers		Non-health benefits	Wider indirect benefits beyond child health
		Trust (facilitated uptake)	Hierarchical trust (f)	Caregivers sought government and medical experts to sanction medication
			Trust in the status quo (f)	CHWs are a conduit of that trust
			A trust learnt though experience (f)	Learnt trust and positive testimony may encourage others
Experiences and attitudes concerning the concept of SMC	Blood testing	Understanding of Chemoprevention and Treatment Philosophy	Poor recall despite experience	Purpose of SMC could be made more clear and acceptable
	Understanding SMC		Medical testing seen as a precursor to any medication (b)	Caregivers conditioned to RDT and medication to treat versus medication to protect
	CHWs’ role in SMC trial		Medication for healthy children a difficult concept (b)	Challenge asking caregivers to re-prioritise their time
Experiences and attitudes concerning the regimen of the chemoprevention, in particular the extended period of SMC delivery	SMC reported dosage	Access to Medication and Dosing Regimen (was sometimes challenging)	Three-day regimen may be difficult to follow (b)	Close supervision of consumption and /or a single day regimen may aid uptake
	SMC 3-day adherence ease and motivation		Five monthly cycles acceptable	Desire to collect SMC
	SMC 3-day adherence challenges and reasoning		Access (b) & (f)	Limited communication of access and restricted modes of access to medication
	SMC 5-month adherence ease and motivation			
	SMC 5-month adherence challenges and reasons			
Preferred place of administration of SMC	Proposed SMC law in Ghana	Preferred distribution method	Caregivers and CHWs give little support for child welfare clinics	Multiple mechanisms of delivery will support users’ needs
	Community meeting point—merits		Caregivers and CHWs give more support for community gatherings and home visits	Better communication and multiple distribution methods would support uptake
	Community meeting point—challenges		CHWs propose community kiosks	Participants suggested meaningful ways to improve delivery and administration including Community Kiosks staffed by CHWs.
	Community weighing centre—merits		CHWs to be trained	
	Community weighing centre—challenges		Finances required to resource CHWs to support delivery	
	At home–merits			
	At home—challenges			
Other	Questions for the Interviewer at the end of interview	*-*		

NB: [(f) = cited facilitator of uptake and (b) = cited barrier of uptake)]

### Reflexivity

To reduce the impact of language, gender and cultural barriers, the field researchers were local female researchers. To deepen insights and ensure findings reflected the research context, in situ analyses were regularly reviewed by study team members who had undertaken the data collection.

### Ethical considerations

Both the RCT and this qualitative sub-study, including the consent procedures, were approved by the ethics committees of the University of Leeds [Ref HSLTLM/11/034], the London School of Hygiene and Tropical Medicine (LSHTM) (Ref 6194) and Komfo Anokye Teaching Hospital / School of Medical Sciences, Kumasi [Ref: CHRPE/AP/117/12]. LSHTM was the trial sponsor. Consent was initially sought from participants at the beginning of the study. Information about the RCT and qualitative sub-study was read and explained to the participants using the participant information leaflet. Written consent to participate in the RCT and qualitative study was then obtained and documented on pre-prepared consent forms using participants’ signature or thumb print. Participants were given a participant information leaflet to retain which detailed what they had consented to including a potential invitation to participate in interview and discussion at the end of the RCT. In appreciation of the lag between the beginning of the RCT and the qualitative sub-study, verbal reconfirmation of consent was sought from all participants and recorded on audio files before participation in IDIs and FGDs. All participants were anonymized in transcription and, once transcribed, all audio data was deleted.

## Results

There were 6 major themes that came out of the study, namely malaria literacy, perceived influence of SMC, trust, understanding of chemoprevention and treatment philosophy, access to medication and dosing regime, and preferred distribution method (see Table 2). Within these major themes were sub-themes which summarise our interpretation of the data and which are discussed in detail under each major theme.

### 1. Malaria literacy

Knowledge regarding malaria was highly varied amongst respondents. The term malaria (in English) was recognised by all caregivers and was often interchangeable with fever. Malaria fever, sun fever, cold food fever, and hard work fever were agreed by all in one FGD to be the same fever (G1). Additional local names were also used including ‘etiridii’ (CHW1, H1), ‘ebunu’ (2A, 2B, 2D, F2, H1, G2), and ‘yare fufuo’ meaning ‘white-disease’ (H1).

Reported physical symptoms attributed by caregivers to malaria included fever, chills, vomiting, diarrhoea, yellowish palms and yellowish urine, feeling weak and restless, bitterness in the mouth and loss of appetite. CHWs also reported children may have joint pain, refuse water, experience a loss of weight and fatigue and associated these with malaria. One caregiver explained it ‘can cause your blood to reduce’ (H1) as a result of vomiting.

Almost two thirds of caregivers from villages with no HCF attributed malaria to a single cause: the mosquito bite. In contrast four of the five respondents with a HCF considered multiple aetiologies for malaria including the mosquito in addition to any of the following: mosquito eggs on food, exposure to extreme heat or a lack of sun, house flies, food preparation and consumption, dirty water, personal hygiene, household hygiene, poor diet or volume of consumption.

Most caregivers (in IDIs and FGDs) were aware of their heightened malaria risk during the rainy season, learnt through distinct periods of increased malaria within their community during changes in the weather, nature’s response to the season (F3) and changes to the farming environment (H1, G1). Three caregivers associated malaria with the ‘sunny season’ (1C, 3E, 1B). FGDs revealed further support for this (F1, G1, H1) explaining a reluctance to sleep under a net (F1). The term malaria was interpretable and may be linked to the believed causes of malaria.

(H1) ‘..when children walk in the sun during the dry season, by the time they come back they would have become weak and leads to malaria.’

When asked about groups more at risk of getting malaria, all participants replied “children”. Three respondents with no HCF also identified pregnant women, and another five respondents also identified adults.

(G2) ‘When you go to the hospital, you realise that there are a lot of children and pregnant women compared to adults. You hardly see adults, except for the elderly (>70years (as defined by H1)) but there are a lot of children.’

Malaria prevention practices reported by caregivers to protect the family involved diligent bed net use (shaking bed net out, early sleeping), protective clothing, closed ventilation indoors, and repellents inside the home, and ‘good environmental hygiene’ to manage ‘filth’, ‘dirt’, and ‘standing water’ outside the home. Caregivers’ knowledge of prevention practices reflected the messages communicated by volunteer CHWs.

CHW 1: Respondent I. ‘We advise the parents to sleep in the nets, to sleep early, and the children less than five years to sleep early. The pregnant women should also sleep early because they are prone to having very severe malaria. We have to weed our backyard and also we should not throw empty tins around. Instead, we can bury them so that the rains cannot collect in them. All gutters should also be de-silted and drained so that they do not breed mosquitoes.’

These practices require caregivers to take personal responsibility for prevention of malaria which both empowered and frustrated caregivers, who reported that they offer little in terms of flexibility during evening routines and do not prevent a child from getting malaria if bitten by a mosquito. As such, malaria remains a constant concern.

(H1) ‘..what worries a bit is that because the mother is still awake, the child will not go to bed so even if you put on the protective clothing the mosquito will get some part of the body to bite.’

(1A) ‘For the children, I cannot really tell how to protect them because children are very difficult to control. In the evening, whilst you are inside, he may be outside. If he is asleep, a parent can place him under the treated net but when you are outside working, he may also be outside with you and during that time too the mosquitoes may bite them and so by the time you go to the room to sleep, he might have already been bitten by the mosquitoes.’

Knowledge of malaria was not identified as an important determinant of SMC uptake, because malaria literacy varied within uptake groupings. In communities with a HCF, despite the opportunity for enhanced knowledge on health matters through contact with health workers, caregivers lacked clarity over the causes of malaria. Nevertheless, even amongst caregiver respondents who knew malaria’s true cause, or were specifically aware of children’s vulnerability to malaria during the rainy season, uptake of SMC remained varied, indicating that malaria-related health education may not improve uptake of SMC.

### 2. Perceived Influence of SMC

Caregivers reported SMC had positive health effects on their children. Caregivers who took four or more monthly doses of SMC reported positive health effects (including no malaria, reduced headaches and no sickness, ceased diarrhoea and no high body temperature) and were motivated by these benefits to continue with taking the medicine (2E, 1A, 2A, 1C, 3B, 2C, 3E, 1C).

(2A) ‘Since I gave her the medicine, I saw a change in her condition and that was my motivation.’

(1D) ‘We are grateful for this drug; you should thank them very much for us. It has saved our children from sicknesses so they should continue with the programme and make the medicines available to the children.’

Caregivers who took fewer than four monthly doses of SMC also noticed positive health responses (including no or reduced episodes of malaria, reduced appetites and regained strength) but despite realising these positive effects, these caregivers had sub-optimal uptake (1B, 2B, 3B, 1D, 2D, 3D) suggesting other factors that influenced uptake. Reasons reported are discussed in Section 5 and include complexity of the medication regimen and access.

Three caregivers, two of whom took four or more monthly doses of SMC, reported temporary negative health effects including high body temperature, yellow urine and vomiting. Negative health effects did not stop some caregivers continuing with the medicine but may have stopped one caregiver from taking the medicine believing her child had fallen ill after consuming it (hearsay reported by 2B).

(2B) ‘A lady attested to the fact that her children fell ill after taking the medicine, so, she stopped coming for it. My child did not have any problem with the medicine.’

Caregivers reporting they had continued giving their children the medicine, despite perceived negative health effects, did so after receiving supportive counsel and encouragement from wider family and CHWs. Informed and supportive assurances from respected peers appeared to counteract some negative influences and may have aided uptake.

(1A) ‘His urine was very yellow so I went to see my grand-father and he asked me to keep on giving him the drug and the sickness will go. And when I continued giving him the drug, I did not see anything again.’

(3B) ‘I was not scared about my child vomiting…my mother motivated me; she said maybe the child had a lot of malaria parasites in her system that is why she is vomiting.’

Caregivers reported other non-health benefits associated with SMC not related to child health. When a child was well, caregivers did not need to travel to the hospital or drug store, nor pay for treatment and laboratory costs. Their reported productivity–spending time working away from home or working on domestic chores at home–also increased. This resulted in time and financial savings and reduced anxiety around child health and malaria. One caregiver indicated her son of ‘school’ age (i.e. 3–5 years old and attending kindergarten) had improved health, suggesting other possible indirect benefits.

(1C) ‘This one (my son) goes to school and always complained of headaches but ever since I started giving this drug, he has not complained about the frequent headaches he used to have so I know it has helped him a lot.’

(2D) ‘…it can give a measure of freedom to parents… as the burden of malaria on parents is too much.’

### 3. Little understanding of chemoprevention

Caregivers found the concept of SMC (chemoprevention) difficult to understand and many could not freely recall ‘any protection medicine for any group of people in Ghana to prevent malaria’, despite their experience with intermittent preventive treatment (a malaria prophylaxis) during pregnancy, and their children’s participation in (and in some cases optimal uptake of) SMC. Some caregivers reported the protection of children from malaria consisted of regular blood checks for parasites and subsequent treatment.

(1A) ‘I cannot protect them unless there is a medicine that can reduce the malaria if the child has it.’

Most caregivers (except 3B) required detailed explanation of the concept of ‘protection’ versus ‘treatment’. This helped some caregivers recall SMC when asked if there was a medicine to protect children under five (2A, 1B, 2B, 3E). Explanation of preventive treatments taken during pregnancy enabled interviewers to help others understand the difference between malaria treatment medication and malaria prevention medication (1A, 2D, 1E). All respondents with a HCF and only a third of respondents without a HCF could, after being prompted, recall SMC and understand its function. Some respondents with sub-optimal uptake understood SMC, suggesting other factors that influenced uptake.

Despite being enrolled in the SMC programme some caregivers remained unclear about its purpose (1C, 2C, 1D, 3D, 2E). A few of these caregivers indicated they would welcome a conceptual medicine like SMC. However caregivers’ theoretical intentions may not reflect behaviour in practice.

(1D) ‘Wherever that medicine is, they should get us some of it to protect our children from malaria.’

Caregivers’ lack of understanding of the concept of SMC is further explained through their opinions around taking medication in the absence of medical testing. Caregivers had mixed reasoning as to the purpose of the initial blood test and the absence of subsequent blood tests and these reasons may have influenced uptake of SMC in some caregivers; some caregivers were cautious in taking SMC without additional testing. Where caregivers continued to accept subsequent doses of SMC some reasoned it was offered as a result of the child’s initial blood test.

(1C) ‘in my opinion, if the child does not have the malaria parasites and takes the (SMC) medicines, it will bring problems to the child.’

(2C) ‘maybe they have seen some sickness in the blood and they made an announcement that we should all come here. So we came.'

Without the blood test CHWs sometimes experienced difficulty in explaining to caregivers why their children should take medicines if they are not sick. Instead some caregivers prioritised their time elsewhere e.g. attending to their work (CHW2). CHWs reported trying to incentivise caregivers by reminding them about the blood test at the end of the rainy season to allow them to establish if the medicine had worked.

(CHW1) ‘They asked us about the results of the first blood test that was done and wanted to know what was wrong with their children. It was difficult for us to answer so we told them that it had been sent to bigger machines to do the test and so later we would hear of the results. And that at the last dosing too, their blood will be checked again to compare with the first one to see if the medicine was helpful. So this is what we told them to encourage them to come for subsequent doses because they wanted to know why the test was done and then the child was given medicine.’

These reactions suggest caregivers’ understanding of chemoprevention varied amongst respondents and, as with malaria literacy and the observed effect of SMC on a child’s health, no clear differences were observed between those with and without optimal uptake.

### 4. Trust facilitated uptake

Caregivers who took and continued to take the medicine did so because they entrusted others in the ‘protection’ of their child. Hierarchical trust in the medical profession and the government, where medical and societal rules prevent harm being done to another [13], is one example of trust.

(1C) ‘You are the doctors and so … if you do not check the blood and you say I should give the medicine to my children I will go ahead and do that.'

(2D) ‘(They would not) give medicine to children with the aim of harming them.’

(3E) 'If you do not accept it you don’t have any medication for the child, so you have to accept it just as you have been given.’

For CHWs and caregivers who understood chemoprevention, it was the combined processes of SMC being ‘sanctioned’ by the government, the experience of taking medication despite no further blood tests, and subsequent observations of no malaria that convinced participants ‘the medicines were to protect the children from getting malaria not to treat the malaria’ (CHW1). With this shared learning experience (a learnt trust) many of the caregivers continued to obtain SMC for their children.

(2B) 'I will allow my child to take medicine. Since (the medicine) does not kill.’

(3B) ‘You need not take medicines only when you are ill.’

Despite caregivers’ undeveloped understanding of the purpose of the SMC, their understanding learnt through experience and shared testimony may have created a snowball effect and triggered other caregivers to trust the programme.

(CHW2) ‘..they have come to believe in the medicine so much… those who were not part are now very serious and want to join in.’

Comments like this lead us to believe that SMC programmes may benefit from supportive testimonies of caregivers who have previously received SMC, and if it is made clear government and medical experts sanction that SMC.

### 5. Access to medication and dosing regimen

Caregiver respondents with optimal and sub-optimal uptake of SMC reported no challenges associated with the SMC three-day regimen, were able to follow the regimen (3B), and reported it was not difficult to administer to the child.

(3E) ‘If they say you should follow the directives and you don’t follow it, the medicine may not work.’

(1C) ‘No it was not difficult to administer the medicines to them.’

However during household visits after the third day of each monthly SMC dose, the CHW estimated (based on some caregivers having tablets remaining) that 10–20% of caregivers had not given day two and day three of the monthly cycle (CHW 1). This suggests, as seen elsewhere, that the three day per month dosing regimen may have been difficult for some caregivers to follow.

The five continuous monthly cycles were considered by all to be well spaced and easy to follow. Caregivers were typically unaware of how many monthly doses they had collected but believed they collected doses when informed by CHWs. CHWs reported that caregivers’ uptake was influenced by ineffective announcements to collect the medication where fixed-point delivery was used, allocation times (1E) and absence of caregivers from home during home delivery.

(1B) ‘I am not sure of the number of times I came for the medicine. I did come till they stopped coming to distribute the medicines but I’ve not checked the number of times.’

(1B) ‘Yes she brought some to my home but she missed me.’

(2B) ‘Monthly intervals are spread out enough and so it is not disturbing.”

(2D) ‘I always come here for the medicine when called so maybe I did not come for it because I was not called.’

Other reported barriers to uptake were having to walk to collect medication particularly during hot days, the perception of the study being for research only rather than personal benefit (CHW1), forgetfulness and laziness.

The fact that some caregivers were found to have not administered some tablets to their children highlights the need for CHWs to check and encourage caregivers to administer the full dose. CHWs proposed an extended seven month period to accommodate children in rural areas with no HCF, suggesting May through November (CHW2). This proposal was also supported in the trial, with evidence that SMC started too late in 2012 relative to the start of the long transmission season [11].

### 6. Preferred distribution method

Respondents were asked to comment on the distribution methods used to administer the SMC during the trail. They were asked about their preferred method in addition to their opinion on the hypothetical distribution of SMC through existing Child Welfare Clinics (CWC). Respondents also identified a fourth potential distribution method; the creation of local kiosks.

#### Child Welfare clinics (CWCs)

CWCs were identified by some respondents as a good place to administer SMC to children between the ages of 0-5years whose mothers attend regularly. Here, it was believed trained medical staff had time to educate attendees, monitor child development, and administer vaccinations.

(3E) ‘Lately they have added one injection at one and half years and the parents bring their children for it.’

Despite having the infrastructure and staff expertise to administer SMC to all children under five years, this option received most objections. Some caregivers had never been to the CWC, or had stopped going after the child had received its last injection around nine months (3B) or had outgrown the ‘weighing’ (2E). Some also found the nurses unapproachable (1D and 2B).

From the caregivers’ perspective, the addition of an SMC programme to existing CWCs had little support given current reluctance towards the continued use of this service after children reached nine months of age. These findings suggest that additional support to capture caregivers who did not or could not attend the CWC would be required.

#### Community gathering

Community gatherings were preferred by caregivers who had become accustomed to this method during the trial (1C), or who preferred medication administered by medical staff (3D). Some felt empowered by actively collecting the medication at the community gathering rather than waiting for a home delivery that ‘could be delayed’ (1B, 1D).

Not all caregivers heard about or were able to go to the public place announced and so this approach may have failed to reach some participants.

Aspects such as being close (3D) to the community centre, being better advertised, having enough water supplies to consume the medication–‘here the pipe is only one’ (2A)–and exposure to medical staff would support the administration of SMC at community gatherings.

#### Household delivery

CHWs made announcements for caregivers to gather and collect SMC and then delivered SMC to children in households whose caregivers did not attend. Caregivers unable to collect medication appreciated the local (1C), explanatory (1C) and family-focused delivery (3B) offered by CHWs. This mechanism extended uptake amongst caregivers who were not reached by fixed-point delivery. CHWs would be unlikely however to successfully deliver SMC to caregivers who were frequently absent from home because of work commitments (e.g. farm workers and traders (3D)). Visiting homes at the close of the day may be an alternative approach but presents its own challenges.

The CHWs believed they were more suitable administrators of the programme than the CWC given their extensive knowledge of caregivers in the community and flexible working hours and outreach capabilities. CHWs preferred full responsibility for the distribution of the medicine so they could reach all households in a ‘systematic way’.

One caregiver preferred the medicine be administered by medical staff (3D) but most were agreeable to administration by ‘trained’ CHWs. In some cases the CHWs were requested to administer the medication to the child; either the caregiver had difficulty in getting their child to take the medicine (3D), or simply because the caregiver entrusted responsibility to the perceived ‘experts’. The CHWs accommodated the additional responsibility and administered the medicine using a combination of gentle coercion, dissolving the medicine in water, and a ‘toffee’ (CHW1 and CHW2) to motivate children who had difficulty in swallowing the ‘bitter taste’ (CHW1).

Many households were remote and difficult to access in bad weather (3E). This approach on its own would require a large and resourced task force to deliver, administer and follow up with all households (1B, 3B). An agreement to leave medicine with non-primary caregivers may support delivery of medication when primary caregivers are not home (3D), and medical training of CHWs could offer reassurance to caregivers and further increase uptake (3D).

Caregivers whose children had optimal uptake identified the community gatherings and home delivery as their preferred mode of administration given the flexibility of these approaches and respect for the medical staff and CHWs. Where a preference was stated, caregivers whose children had suboptimal uptake also preferred these modes. For both modes of administration, additional supportive resources relating to advertising the gatherings, and remuneration of the CHWs specifically for SMC (in addition to their duties in HMM), would support delivery of medication.

#### Community kiosks

It was proposed that local kiosks could be permanently stationed in the community and would act as a reminder and distribution service. The kiosks would be distinct from the CHWs’ homes and ‘belong to the community’ (CHW2) triggering passing caregivers to collect the medicine.

(CHW2) ‘Like lotto operators, everyone has their kiosk so on Saturdays everyone knows that if they go to the kiosks, they can stake their lotto. We should get permanent posts like small kiosks in the communities which are centres for children under five for everyone to know. When the women pass by, they will see the kiosks and be reminded of coming to receive the medicine for the children. They will know that there is a particular group of people at this place who give medicines for free so will come.’

(CHW2) ‘The kiosks, as he said, are important such (that) we can dedicate specific weeks for receiving the medicines. I think in this way, they will come for them.’

#### Incentives

It was reported that incentivising caregivers would support collection of SMC. Incentives would take the form of a gift of tokens or small gifts such as soap. CHWs indicated that the household or community administration of the medicines would further be improved if they too were motivated through the following means: a formalised contract, remuneration (such as 200 Ghana cedis/month), sanctioning from the government of their work and responsibilities, and transport aids such as a bicycle or motorbike to cover the larger communities or geographical areas.

## Discussion

SMC has the potential to avert a large number of clinical malaria episodes during the malaria transmission season [14], but only if SMC drugs reach caregivers and are correctly administered. It is therefore essential to understand the acceptability of the extended SMC programme and identify facilitators and barriers to caregivers’ uptake of SMC.

### Acceptability

The length of the five month SMC programme was acceptable in principle to caregivers, albeit many could not recall how many cycles they had had. Whilst caregivers reported the 3-day per month regimen was also acceptable, in practice the full dose was not always consumed as evidenced by CHWs during home visits. The complexity of the regimen may have influenced uptake and subsequent efficacy of the SMC [11]. It was widely recognised that a one day regimen would be preferable for SMC and would reduce the burden of drug administration on caregivers.

Whilst the SMC programme sought to alleviate the risk of malaria in children it may not have met the perceived needs of caregivers [15]. Caregivers believed there was no need to medicate children who were well: conveyance of the reported non-health benefits associated with SMC may widen appreciation of the medication. Experience of chemoprevention during pregnancy had not accustomed caregivers to medication against their behavioural norm, i.e. to medicate without clinical symptoms, testing and diagnosis, particularly against a backdrop of Test Treat and Track for malaria [16]. A patient’s own decision-making around treatment can be complex and challenging [17], as can that of a patient’s or child’s caregiver [18] despite being ‘risk aware’, and motivated towards their welfare. Values such as a caregiver’s priorities (work vs. ensuring a child is well), life philosophy (treat to protect), self-efficacy (perceived ability) and background (malaria is a risk presented to all) are all likely to contribute to decision-making [17]. A combination of caregivers’ physical access to SMC medication, self-belief in being able to effectively protect a child from malaria [19] and perceived norms around malaria prevention are all likely to have influenced caregivers’ level of uptake.

### Facilitators

There did not appear to be major differences between caregivers of children with optimal and sub-optimal SMC uptake in terms of their knowledge of malaria, their perceptions of the effect of SMC on a child’s health, nor their understanding of chemoprevention. In this study it was not possible to identify how or if knowledge influenced respondents’ behaviour (i.e. optimal or sub-optimal uptake) [12, 20], but other facilitators of uptake were suggested.

Reported facilitators of uptake included caregivers’ hierarchical trust and respect for medical experts and CHWs, supportive community networks that alleviated concerns about chemoprevention, and learnt trust through experience of the SMC during the programme.

Caregivers had varied knowledge of malaria and its causes, and almost no knowledge of chemoprevention despite the SMC programme and generally high use of Intermittent Preventive Treatment in Pregnancy (ITPp) in the area [21]. An entrenched culture of trust can occur where health awareness is poor, as seen in these villages. Loyalty, belief in the competency of and respect for the medical professionals are dimensions of trust previously identified in resource poor health care settings [22]. Caregivers’ comments imply an unwritten covenant between government, medics and CHWs that protects the vulnerable. The caregivers’ trust exhibits pre-defined expectations relating to non-exploitation and acquiescence. Caregivers’ trust in experts’ knowledge reduced the two-way participation in informed decision-making seen in ‘developed countries’ [23–25] and has been identified elsewhere as a reason for initial adoption of a new preventive medication [15, 26]. However, caregivers had some concerns over the absence of regular blood tests, as they had become used to existing provision of RDT. Extending caregivers’ trust to include chemoprevention is one area where trust could be improved. CHWs may be key in offering that sense of social connectedness and familiarity, which increases confidence, and trust in a programme [22], particularly important given caregivers’ varied health literacy and lack of autonomy in health.

Some caregivers benefited from talking about the medication [21] to family members and CHWs who reinforced the worth of the medication and gave them reassurance to continue. Positive testimonies of the protective effect of the medication created a snowball effect and triggered requests from the wider community to join the programme. Sensitisation meetings that promote community understanding and involvement in drug administration programmes can maintain and increase uptake [27]. Recruitment and advocacy from clinicians such as hospital workers (particularly those involved in IPTp and malaria treatment), drug stores and third party local groups, and previous users of SMC would not only build buy-in [28] amongst the health sector but offer wider more holistic support to the community.

### Barriers

Barriers to uptake related to challenges around access to medication and dosing regimen to reflect the preferred distribution method and the treatment philosophy of caregivers and CHWs. Caregivers were less engaged in the nuances of malaria and preventive medication. Rather, access (communication and administration) to chemoprevention through the use of flexible and accessible modes of administration (through home visits and fixed point provision respectively) appeared preferable; other studies suggest this may give greater uptake [5] than offering access through existing CWCs, whose use can be limited [29]. Caregivers may have disconnected with the SMC programme as a result of inconvenient access to the SMC. Restricting communication of the programme to participants to the use of local FM stations, coupled with caregivers’ work commitments reduced attendance at community gatherings. Community gatherings acted as a social and supportive opportunity to share experiences but required better advertising to improve attendance and uptake. Household delivery was a more flexible method as CHWs could operate flexible working hours. However appropriate transport, training and remuneration would be required [15] to enable CHWs to access often hard to reach households. Levels of optimal uptake may increase if the household delivery strategy allowed medication to be left with any adult of a household in the absence of the primary caregiver. The two modes could operate exclusively or jointly and be tailored to each village relative to the funding and training needs of CHWs, and the distances and terrain covered by caregivers and CHWs. Pop-up community kiosks situated in communities could also encourage and enable caregivers to collect the medication every month during the malarial season.

Caregivers could comprehend malaria treatment medication much better than malaria prevention medication. Caregivers had, independently of the trial, been exposed to routine health education whereby the reasons for children’s ill symptoms were screened using diagnostic blood tests that revealed the presence or absence of malaria parasites prior to treatment. During the trial, blood samples were taken from participating children at the beginning of the study, as part of a cross-sectional survey prior to the first cycle of SMC. All children (apart from those who were unwell and who were referred for treatment) subsequently received SMC or matching placebo. However, encouraging subsequent uptake of SMC in the absence of further blood tests each month evoked discussion amongst caregivers who appeared conditioned to take further malaria medication only after further testing. Whilst appropriate diagnosis and treatment is important [22], the RDT programme may have acted as a barrier to sustained uptake of chemoprevention amongst respondents; and this confusion about diagnostic testing before treatment and no testing before prevention may have been compounded by the use of diagnostic testing at the first cross-sectional survey.

## Recommendations

The study identified the trust these communities place in the medical profession and the role this plays in SMC uptake. The medical profession, in conjunction with local government, must reflect how best to constructively use this trust to support optimal uptake. Cantey [30] recommends piloted data such as this is used to inform and educate mass drug administration programmes. A targeted, clear and timely pre-SMC educational campaign that reflects the facilitators and barriers to uptake amongst caregiver respondents (Table 3) would sensitise and enable users to maximise uptake and associated health benefits offered by SMC. Such campaigns are being implemented in more seasonal areas of West Africa where SMC is now being implemented at scale.

**Table 3:** Summary of faciltators and barriers to SMC uptake amongst caregiver respondents, collected during a qualitative study in Ghana in January 2013.

Facilitators of SMC uptake	Barriers to SMC uptake
• Trust in and respect for authorities who were seen to sanction and implement the SMC	• SMC programme was incompatible with some caregivers perceived needs, who believed there was no need to medicate children who were not sick and their time was better spent at work
• Proximity to and communication of fixed point delivery (community gatherings)	• Large distances to travel, restricted timings of, and poor communication of fixed point delivery (community gatherings)
• Flexible door-to-door (household) delivery	• Delivery of medication only to primary caregiver during door-to-door visits
• Beliefs that any perceived side-effects of SMC were attributable to the SMC medication treating undiagnosed malaria in the child	• Beliefs that any perceived side-effects of SMC were attributable to the SMC medication harming the child
• CHW supervision and administration of medication directly to the child at home.	• Need to consume all SMC medication over 3 consecutive days within a month.
• Reference to IPTp to explain the difference between malaria treatment medication and malaria prevention medication	• Caregivers found the concept of preventive medication difficult to understand despite experience of IPTp and the SMC programme
• Observation of other caregivers’ participation and their perceived positive health responses	• Belief the intervention was for research only and not routine care
• Reassurance from CHWs and senior family members on perceived side-effects (the basis for a supportive community-based network)	

SMC programmes need to consider 1) developing supportive, accessible and flexible modes of drug administration including door-to-door delivery and fixed point distribution, 2) improving demand for preventive medication including harnessing learnt trust in the medication which caregivers developed through their shared experiences during the programme, and 3) developing supportive community-based networks for users to encourage optimal uptake of SMC.
